# Differential Detection of Genetic Loci Underlying Stem and Root Lignin Content in *Populus*

**DOI:** 10.1371/journal.pone.0014021

**Published:** 2010-11-22

**Authors:** Tongming Yin, Xinye Zhang, Lee Gunter, Ranjan Priya, Robert Sykes, Mark Davis, Stan D. Wullschleger, Gerald A. Tuskan

**Affiliations:** 1 Environmental Sciences Division, Oak Ridge National Laboratory, Oak Ridge, Tennessee, United States of America; 2 Bioenergy Science Center, Oak Ridge, Tennessee, United States of America; 3 The Key Lab of Forest Genetics and Gene Engineering, Nanjing Forestry University, Nanjing, China; 4 National Renewable Energy Laboratory, National Bioenergy Center, Golden, Colorado, United States of America; University of Massachusetts Amherst, United States of America

## Abstract

In this study, we established a comprehensive genetic map with a large number of progeny from a three-generation hybrid *Populus* intercross, and phenotyped the lignin content, S/G ratio and 28 cell wall subcomponents both in stems and roots for the mapping individuals. Phenotypic analysis revealed that lignin content and syringyl-to-guaiacyl (S/G) ratio using pyrolysis molecular beam mass spectroscopy (pyMBMS) varied among mapping individuals. Phenotypic analysis revealed that stem lignin content is significantly higher than that in root and the quantified traits can be classified into four distinct groups, with strong correlations observed among components within organs. Altogether, 179 coordinating QTLs were detected, and they were co-localized into 49 genetic loci, 27 of which appear to be pleiotropic. Many of the detected genetic loci were detected differentially in stem and root. This is the first report of separate genetic loci controlling cell wall phenotypes above and below ground. These results suggest that it may be possible to modify lignin content and composition via breed and/or engineer as a means of simultaneously improving *Populus* for cellulosic ethanol production and carbon sequestration.

## Introduction

Wood is a heterogeneous, hygroscopic, cellular and anisotropic material composed of three major components: cellulose, hemicellulose and lignin. Cellulose and hemicellulose are polysaccharides, comprising 65%–75% of the dry mass of wood [1]–[2]. Lignin, a phenolic polymer consisting of three alternate hydroxycinnamyl alcohols precursors [3]–[6], embeds the polysaccharide matrix giving stiffness and cohesiveness to the woody tissue and providing hydrophobic surfaces needed for water transport [7]–[8]. Highly lignified wood is rigid and durable and therefore a good material for many structural applications. However, lignin must be removed in the process of manufacturing high-quality bleached paper and in bioethanol production [9]–[12]. Thus, the amount of lignin impacts cell wall structure and function, as well as the technological value of raw materials [3], [13]–[15]. For simultaneous applications directed towards improved pulp yields, enhanced bioethanol production and increased carbon sequestration, it would be desirable to reduce lignin in the harvested stem while increasing the lignin content in non-harvested root [9], [16]–[17]. Yet, lignin content in belowground plant structures is not well quantified and its relationship to lignin content in the aboveground organs remains ambiguous.

The biochemical pathway for lignin biosynthesis is fairly well characterized and involves approximately 12–15 enzyme-regulated steps, generally controlling the conversion of aldehydes to hydroxyl, guaiacyl and syringyl precursors [5]–[6], [18]. Lignin content varies by species, across tissues and organs, with developmental age, and by environmental triggers/influences [17], [19]. These responses are genetically controlled and heritabilities for lignin are moderately high. In the last decade, our understanding of the lignin biosynthetic pathway has rapidly progressed to the point where researchers have isolated and cloned several lignin biosynthesis genes and characterized their expression *in vivo* [20]–[24].

Hybrid poplars (*Populus* spp.) are among the fastest growing trees in the world, providing raw material to the pulping industry and having great potential in bioethanol production. *Populus* is the first woody plant with whole-genome assembly and annotation data available [25]. The genome of *Populus* contains evidence of three whole-genome duplication events. The most recent, the *Salicoid* duplication, is found only in members of the *Salicoid* family and is represented in 16,000 paralogous gene pairs. In addition, the molecular clock in *Populus* is ticking at a rate that is 6 times slower than in *Arabidopsis*, and together with the whole-genome duplication, allows for a duplicated molecular preservation of the ancestral genome within the extant *Populus* genome. Together with the availability of a high-density genetic map [26] and integrated physical map [27], *Populus* has been widely adopted as model system for functional genomics studies in woody plants.

In this study, we employed pyrolysis molecular beam mass spectroscopy (pyMBMS) to characterize lignin content, syringyl-to-guaiacyl (S/G) ratio and 28 spectral cell wall subcomponents in stems and roots of a large number of progeny from a three-generation interspecific *Populus* pedigree. By integrating pyMBMS phenotyping, comparative intragenomic analysis, and QTL analysis, we identified genomic regions associated with lignin content in roots and/or stems, mapped the coordinating genetic loci, and provide markers which can be used to enable breeding efforts focused on increased lignin content in roots for enhanced soil carbon sequestration and/or decreased lignin content in stem for improved conversion of lignocellulosic feedstocks to ethanol.

## Results

### Phenotypic analysis

Based on the pyMBMS measurements on 292 progeny in Family 331, the average lignin content across all genotypes was 24.3% in stem and 22.2% in root, representing a higher average in stems. The average S/G ratio values were 2.0 in stem and 1.4 in root; representing a 40.8% higher average ratio in stems. Based on ANOVA results, both lignin content and S/G ratio in stem are significantly (α≤0.01) higher than those in root (Table 1). In addition to lignin content and S/G ratio, we analyzed 28 pyMBMS spectral peaks associated with cell wall subcomponents in stems and roots. Fourteen of these subcomponent peaks were significantly higher in stem than in root; 13 were significantly higher in root. The remaining peak, *m/z* 58 (a nonspecific polysaccharide peak) had similar values in stems and roots. Chemical characterization for cell wall subcomponents for all 28 peaks is listed in Table 1.

**Table 1:** ANOVA and correlation analyses for quantified traits in stem and root.

	Tissue (correlation group, mean)		ANOVA		
Trait (m/z)	Root	Stem	F	*p*-value^1^	Compound name^2^
41	IV, 0.64	III, 0.29	6452.05	0.0000**−	nonspecific fragment from protein or sugar
43	IV, 4.93	III, 2.80	2764.50	0.0000**−	nonspecific peak for polysaccharides
57	IV, 141	III, 130	38.43	0.0000**−	nonspecific peak for polysaccharides
58	IV, 0.73	III, 0.73	1.08	0.3420	nonspecific peak for polysaccharides
60	IV, 1.70	III, 1.35	84.92	0.0000**−	nonspecific peak for polysaccharides
73	IV, 1.94	III, 1.43	221.05	0.0000**−	nonspecific peak for polysaccharides
85	IV, 2.66	III, 2.78	21.05	0.0000**+	nonspecific peak for polysaccharides
94	II, 0.63	III, 0.42	330.58	0.0000**−	phenol
97	IV, 1.19	III, 1.24	9.06	0.0027**+	di or tri substituted aromatic
98	IV, 1.26	III, 1.71	973.50	0.0000**+	2,5-dihydro-5-methylfuran-2-one, furfuryl alcohol
114	IV, 1.37	III, 1.44	11.24	0.0009**+	3-hydroxy-2-penteno-1,5-lactone
120	II, 0.26	I, 0.18	1065.55	0.0000**−	acetophenone, 4-vinyl-phenol
124	II, 1.25	I, 0.87	861.17	0.0000**−	guaiacol
126	IV, 1.36	III, 1.24	143.76	0.0000**−	dimethyldihydropyranone, 5-hydroxymethyl-2-furaldehyde
137	II, 2.01	I, 1.45	774.02	0.0000**−	ethylguaicol, homovanillin, coniferyl alcohol
138	II, 1.39	I, 0.85	1840.53	0.0000**−	methylguaiacol
144	IV, 0.40	III, 0.35	44.25	0.0000**−	1,4-dideoxy-D-glycero-hex-1-enopyranose-3-ulose
150	II, 0.70	I, 0.76	62.30	0.0000**+	vinylguaiacol, coumaryl alcohol
154	II, 0.91	I, 1.45	1887.95	0.0000**+	syringol
164	II, 0.58	I, 0.55	27.94	0.0000**−	allyl propenyl guaiacol
167	II, 1.61	I, 1.87	103.49	0.0000**+	ethylsyringol, syringylacetone, propiosyringone
168	II, 0.76	I, 0.88	176.55	0.0000**+	4-methyl-2,6-dimethoxyphenol
178	II, 0.40	I, 0.47	376.77	0.0000**+	2-methoxy-4(prop-2-enal)phenol
180	II, 1.41	I, 2.12	961.37	0.0000**+	coniferyl alcohol, vinylsyringol, α-D-glucose
182	II, 0.50	I, 0.89	1756.66	0.0000**+	syringaldehyde
194	II, 0.47	I, 0.77	1262.78	0.0000**+	4-propenyl syringol, ferulic acid
208	II, 0.23	I, 0.47	2190.09	0.0000**+	sinapylaldehyde
210	II, 0.54	I, 1.58	2895.33	0.0000**+	sinapylalcohol
lignin content	II, 2.22	I, 2.43	215.61	0.0000**+	lignin (% dry weight)
S/G ratio	II, 1.42	I, 2.01	1093.42	0.0000**+	syringyl to guaiacyl ratio

**Figure 1:**
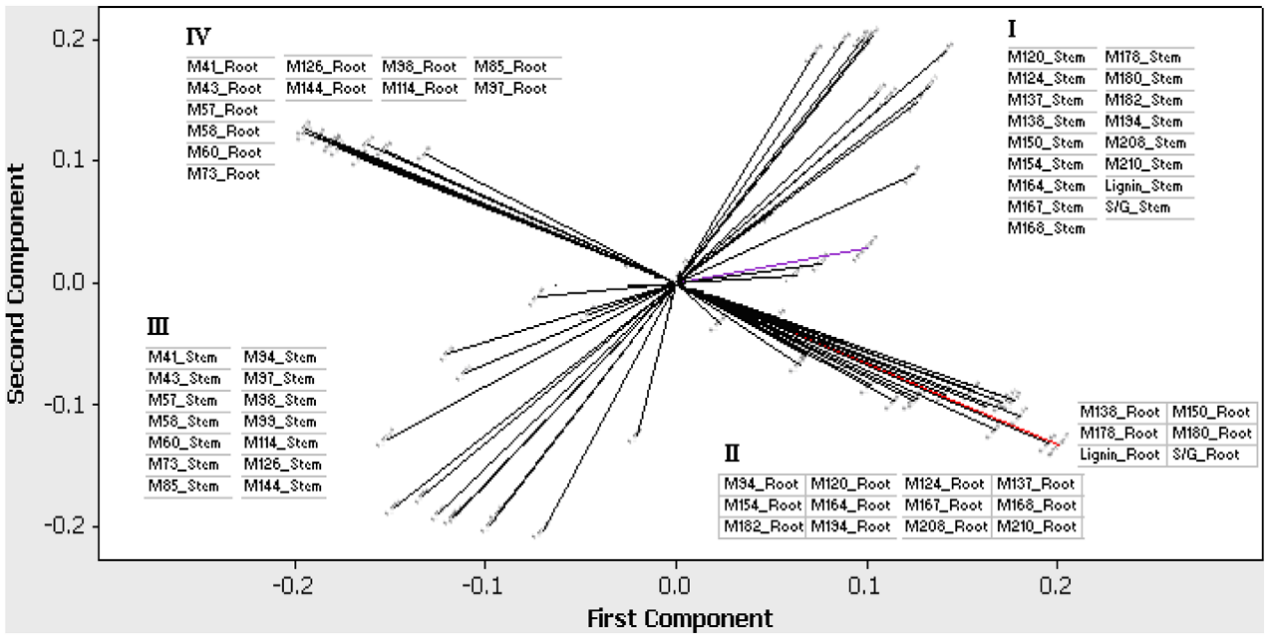
Principal component analysis for all the quantified traits. Note: correlation loadings were plotted for the first two principal components. The red line represents root lignin and the purple represents stem lignin. Subcomponents within each cluster are positively correlated, by contrast, subcomponents in opposite clusters are negatively correlated (e.g., cluster I vs. cluster III, and cluster II vs. cluster IV).

1 * indicates significance at α≤0.05; ** at α≤0.01; the “−” following the “*” indicates less than expected number at corresponding significant level; “+” following the “*” indicates more abundant than expected number at corresponding significant level. Correlation groups were designated according to analyses in Figure 1.

2 “Nonspecific” indicates that the pyrolysis breakdown product could not be definitively characterized and/or may be the result of a combination of multiple breakdown products.

Principal component analysis was employed to explore the multivariate correlations among all the quantified traits. The first and second principal components explained 51.4% of the total variation. Based on the correlation loading on the first two principal components, the quantified traits can be grouped into four clusters. The cell wall subcomponent spectra in each cluster were tissue specific (Fig. 1), i.e., cell wall subcomponents in cluster I and cluster III are stem specific, while subcomponents in cluster II and cluster IV are root specific. It is noteworthy that subcomponents in cluster I are positively correlated with stem lignin; whereas subcomponents in cluster III are negatively correlated with stem lignin. Similarly, subcomponents in cluster II are positively correlated with root lignin; in contrast, subcomponents in cluster IV are negatively correlated with root lignin. Cluster I and II are comprised of mainly polyphenolic-related, pyrolysis-derived products of cell walls and cluster III and IV are comprised of mainly polysaccharide-associated, pyrolysis-derived products (Fig. 1, Table 1).

Based on individual pair-wise correlations (Table S1), the cell wall subcomponent spectra that shows the highest positive correlation with root lignin was *m/z* 180 (a peak related to coniferyl alcohol & vinylsyringol) (r = 0.92, representing 84.6% of the root lignin variance). Spectra *m/z* 73 (a nonspecific polysaccharide peak) was the subcomponent peak that shows the largest negative correlation with root lignin (r = −0.94, representing 87.3% of the root lignin variance). The subcomponent spectra having the highest positive correlation with stem lignin was *m/z* 150 (a peak related to vinylguaiacol and coumaryl alcohol) (r = 0.56, representing 32% of the stem lignin variance). The subcomponent spectra with the highest negative correlation with stem lignin was *m/z* 73 (a nonspecific polysaccharide peak) (r = −0.74, representing 54.5% of the stem lignin variance). It is noteworthy that spectra *m/z* 73 (a nonspecific polysaccharide peak) was the most negatively correlated peak with lignin content in both roots and stems.

From the above analyses, we hypothesize that to lower stem lignin content via transgenesis, genes affecting lignin content should be down regulated in cluster I and up-regulate in cluster III. Alternatively, to increase lignin content in root, genes should be up regulated in cluster II and down regulated in cluster IV.

### Map construction

**Figure 2:**
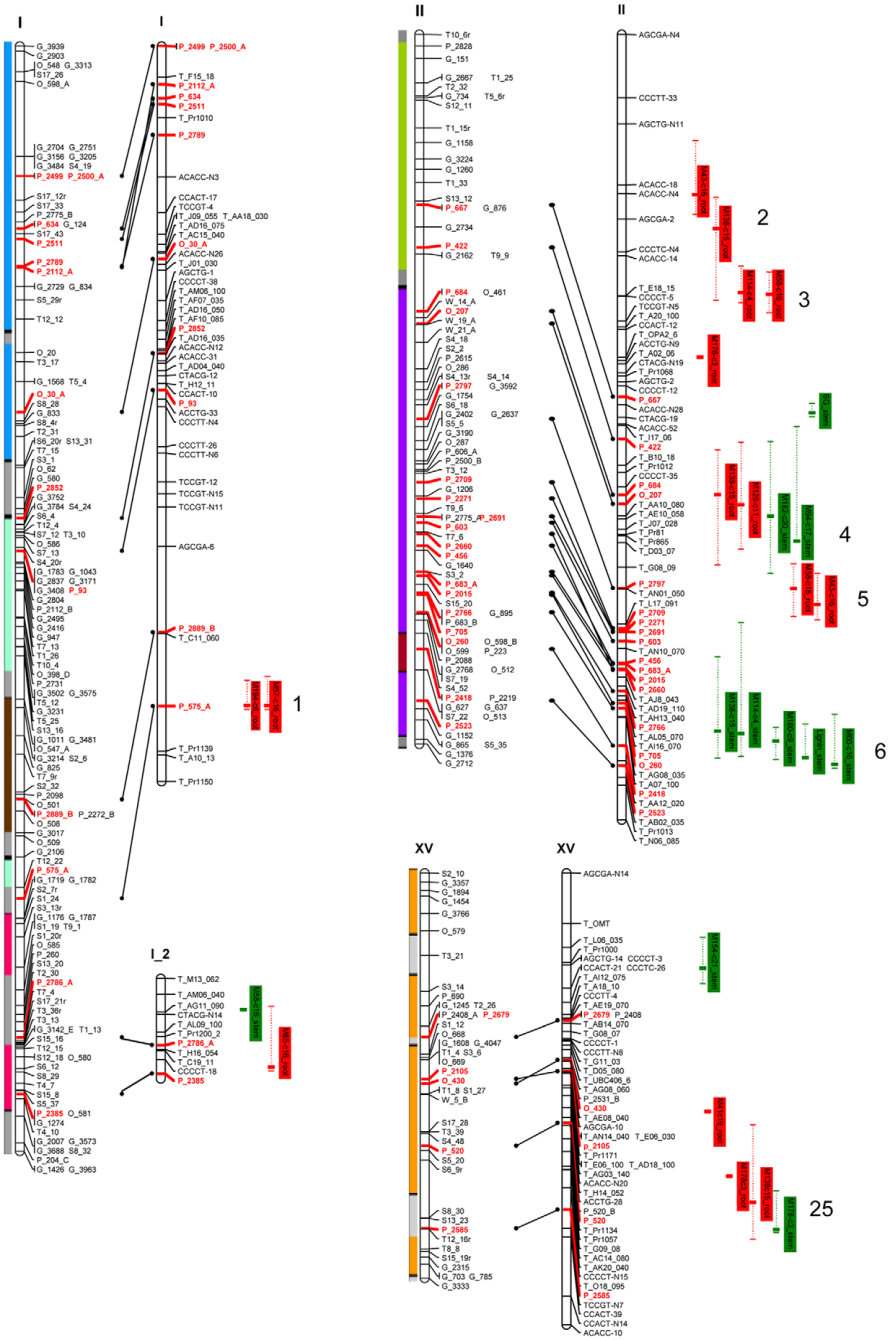
Genetic map and locations of the identified QTLs for Family 331. Linkage groups of the consensus map are on the left and linkage groups of the Family 331 map are on the right. Shared SSR markers between the two maps are indicated with connecting lines. Colored bars on the right side of each linkage group represent the map regions of the established QTL intervals, and the bold vertical bar in each interval marks the position of the LOD peak for each QTL. Bars in green indicated QTLs detected in stem, and bars in red indicated QTLs detected in root. The cell wall components are listed in each vertical and correspond to compound names in Table 1. The colored bar on the left of each linkage groups match paralogous regions in the genome based on Figure 2 in Tuskan et al. [25]. These bars were scaled based on the genetic length of each linkage group from the consensus map.

A comprehensive genetic map containing 848 markers was constructed for Family 331. The overall observed genetic length was 1927.6 cM. The established map consists of 20 linkage groups (LG). The derived linkage groups were subsequently aligned to 19 haploid chromosomes of *Populus* using homology with the *Populus* consensus map [28] and 155 shared SSR markers (Fig. 2). Based on shared marker alignment, the 98% of the mapped SSR markers were colinear with those on the consensus map. We only detected discrepancies in marker order at five loci, possibly related to genotyping errors or covert chromosomal rearrangements in the alternative parental genotypes. Besides the high degree of synteny between the two maps, genetic distance between markers on the two maps was also highly correlated (r = 0.98, p≤0.0001). A recombination rate heterogeneity test was performed to detect marker pairs with significantly dissimilar numbers of crossover events per meiosis in the alternative mapping pedigrees. Among the shared markers, we detected only one marker pair with significant recombination heterogeneities at α≤0.01 and another three marker pairs at α≤0.05. These results suggest that both marker order and recombination frequencies between the shared markers are conserved in the two mapping pedigrees.

### QTL detection

Results of a phenotypic data distribution test and normality transformation are listed in Table S2. Among all 60 phenotypes, 49 were normally distributed or could be transformed into normality. Eleven traits were skewed from normal distribution and could not be transformed and were thus analyzed using non-parametric approaches. The results of QTL analyses are presented in Table S3 and Fig. 2. In total, 179 QTLs were detected for the entire set of cell wall traits. Each trait was associated with 1 to 7 QTLs dispersed among 17 chromosomes of *Populus* genome. No QTLs were detected for 6 of the measured traits and no QTLs were detected on linkage groups XVII and XIX. The percentage of the phenotypic variance explained by the QTLs ranged from 4.3% to 22.9%. The largest QTL (LOD = 6.83) was observed on LG X for the cell wall subcomponent *m/z* 138 in stem, which is related to methylguaiacol and positively correlated with stem lignin content.

It is noteworthy that numbers of detected QTLs on each linkage group varied significantly among the chromosomes. Based on the Poisson calculator, we found that QTLs were significantly (α≤0.01) overrepresented on LG VI, VIII, X and XIV (Fig. 2, Table S4). Recall that the modern *Populus* genome arose from a highly conserved whole-genome duplication event [25]. Thus, alternate pairs of *Populus* chromosomes share high homology and synteny (Fig. 2, e.g., LG VIII and X or LG XIV and II). Comparing all QTLs with their paralogous chromosomes it appears that loss of function and/or subfunctionalization has occurred among the paralogous chromosomes. For example, 37 QTLs were detected on LG X and only 17 QTLs were detected on its homologous regions on LG VIII and only 12 of the 37 QTLs detected on LG X corresponded to the same trait QTL on LG VIII (Fig. 2, Table S4).

### Pleiotropic QTLs and differential detection of QTLs in stem and root

Among the 179 detected QTLs, 108 were associated with root phenotypes and 71 with stem phenotypes. The average percent variance explained was higher for roots than stems. Interestingly the vast majority (i.e., 155 out of 179) of the QTLs detected in this study co-localized with at least one other QTL. Pleiotropy occurs when a single genetic loci influences multiple phenotypic traits. If we establish a LOD peak position within a 2 cM interval as a single pleiotropic genetic locus, then 155 QTLs would co-localize into 27 putative pleiotropic genetic loci, with each locus corresponding to two to 32 traits (Table S5). Putative pleiotropic genetic loci were observed on 16 of the chromosomes. Hypothetically, overexpression of the pleiotropic gene(s) responsible for the QTL effect will increase the content of the positively correlated cell wall subcomponents. Empirically, among the 27 pleiotropic genetic loci, 16 solely contained traits within multivariate clusters and/or traits in opposite clusters (Fig. 1). According to the phenotypic data analyses, strong correlations were only detected for traits within clusters or in opposite clusters. Among the 27 pleiotropic genetic loci, 10 were root specific and six stem specifics, supporting the pleiotropic hypothesis. An alternative hypothesis is that there multiple, independent genetic loci within each QTL interval responsible for the observed phenotypes. There were 13 monotropic genetic loci associated with only root phenotypes and nine with only stem phenotypes. In summary, of the 179 QTLs representing 49 genetic loci, 23 were differentially associated with root phenotypes, 15 with stem phenotypes, and 11 in both stem and root phenotypes.

## Discussion

Stem lignin and stem S/G ratio for *Populus* have been reported in an alternate *Populus* family, Family 52–124 from which phenotypes were obtained from greenhouse-grown progeny in an interspecific pseudo-backcross pedigree [17]. The grandfather of Family 52–124 is *P. deltoides* ‘ILL-101’ and the father is *P. deltoides* ‘D-124’; the *P. deltoides* parent in our study was ‘ILL-129’. The two pedigrees do share the same *P. trichocarpa* grandmother, ‘93–968’. No common QTLs underlying stem lignin or stem S/G ratio were detected between the two studies. Among the four QTLs detected in Family 52–124, the positive alleles of three QTLs originated from *P. deltoides* and only one positive allele originated from *P. trichocarpa*. The detection of QTLs depends on the heterozygosity of the underlying genetic loci, the general genetic background of the parents (i.e., epistasis), and each alleles' affect on the phenotype. We propose that the differences in QTLs detected in Family 331 vs. Family 52–124 is related to the differences in genetic background, heterozygosity and allelic effects among the alternate *P. deltoides* parents used to create the two pedigrees. In our case, the progeny of Family 331 might not have inherited positive alleles from the alternate *P. deltoides* parent. If true, this suggests that the QTLs detected in the two studies would not be orthogonal, but should be additive to each other.

Interestingly, we detected a larger number of QTLs underlying stem lignin and stem S/G ratio than did Novaes et al. [17]. For lignin content in stem, only one QTL was detected in Family 52–124; whereas there were four QTLs detected in our study of Family 331. For stem S/G ratio, three QTLs were detected in Family 52–124, compared to five QTLs in Family 331. This difference may be due to genetic differences between the two pedigrees (noted above). Alternatively, the F_2_ pedigree used in our study potentially increases the number of segregation events and creates larger differences in allelic effects than does a backcross pedigree. In addition, the analytical methods can also affect the numbers of detected QTL. In the Novaes et al. [17] paper, QTLs were analyzed based on pseudo-testcross strategy. *Populus* is an outbred set of diploid species with up to four alleles at each locus. The pseudo-testcross approach pools alleles at each locus into *a* and 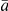 (a pool of allele b, c and d). Although a pseudo-testcross strategy facilitates map construction, it decreases the power for QTL detection. In our study, QTLs were detected based on cross-pollinated mating types, the effects of all alleles were taken into consideration, and thus, a larger number of QTLs may be anticipated.

It is becoming apparent that most eukaryotic genomes contain numerous duplicated genes, many of which appear to have arisen from one or more whole-genome duplication events [29]–[31]. In *Populus*, cytological studies revealed that all extant species existed in the diploid form with a haploid number of chromosomes equal to 19 [32]–[33]. However, the *Populus* genome sequencing project revealed three separate genome-wide duplication events contained within the *Populus* genome, with 84% of the all genes within the genome arising within the most recent event [25]. In theory, there can be extensive loss and/or subfunctionalization of duplicated genes following genome doubling [29]–[31]. In this study, we found that a number of QTLs and their homologs on paralogous syntenic regions appear to have functionally diverged. For example, there are three paralogous chromosomal regions that contain QTLs in only one to the two paralogs (III vs. I, XII vs. XV and XIII vs. XIX, Fig. 2). Moreover, chromosome XVII and XIX contained no QTLs even though their paralogs contained multiple QTL loci each with multiple co-located phenotypes. The lack of QTLs on chromosomes XVII and XIX may be related whole-chromosome subfunctionalization, chromosome XVII contains a large 5SrDNA segment [33] and chromosome XIX appears to be evolving into a sex chromosome [26].

There are three chromosome intervals where multiple stem and root phenotypes co-locate to single position on the *Populus* genetic map (i.e., LG VI, X and XIV), implying that there may be a gene(s) within each interval that controls general lignin biosynthesis above and below ground. Modifying the expression genes responsible for these phenotypes may cause changes in the whole plant lignin content or composition. Alternatively, there are several QTLs that are stem specific or root specific (e.g., LG I, VI and XIII). This is the first report of QTL for root lignin and the first report of separate loci determining stem and root lignin content. These results suggest it may be possible to modify lignin content or composition above ground and not impact lignin content below ground.

In summary, lignin content varies greatly among species. In *P. trichocarpa* alone, lignin content ranges between 15.7% and 27.8% among individual genotypes [10]. Delignification requires harsh chemical treatments and high energy inputs [34]–[36]. Reduction in lignin content in the stems of harvested trees would benefit the pulp and paper and/or ethanol production industries. In contrast, lignin, as a recalcitrant cell wall subcomponent rich in carbon, provides an opportunity for enhanced long-term carbon sequestration in soils from the non-harvest plant structures [17], [37]. The wide natural variation found in stem and root lignin content reported here provides the possibility for altered lignin content via conventional breeding programs or by modern molecular breeding techniques.

## Materials and Methods

### Mapping pedigree and genotyping

Family 331, an F_2_ inbred interspecific hybrid family, was created through a sib-mating between female 53-246 and male 53-242 of the F_1_ hybrid (T×D) Family 53, and was grown in three clonal blocks under fertigation in Wallula, WA for five years. Family 53 is a cross between a female *P. trichocarpa* (93-968) and a male *P. deltoides* (ILL-129). To establish the genetic map for Family 331, a total of 848 markers were generated. Among these markers, 210 were SSRs, which were genotyped with 310 progeny at Oak Ridge National Laboratory (ORNL). Besides the SSRs, 208 AFLP markers were generated with 184 progeny. SSR and AFLP marker generation, genotyping and nomenclature were as described in Tuskan et al. [38] and Yin et al. [28]. The remaining 430 markers consisted of RAPDs and RFLPs, which were genotyped with 92 progeny [see 39 for details]. Map construction and recombination heterogeneity test were conducted by JoinMap 3.0 [40]. The RAPDs and RFLPs were only used to integrate the genetic map and were excluded from QTL analysis due to the small population size and high frequency of missing data.

### Phenotyping and phenotypic data analysis

Lignin content, S/G ratio, and cell wall subcomponents were phenotyped using pyMBMS at National Renewable Energy Laboratory, USA as described in Tuskan et al. [41], Davis et al. [42] and Sykes et al. [19]. Pearson product-moment correlation coefficient (*r_xy_*) was used to estimate the correlation of trait *X* and trait *Y*, which is formulated as below:
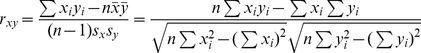
(1)where 
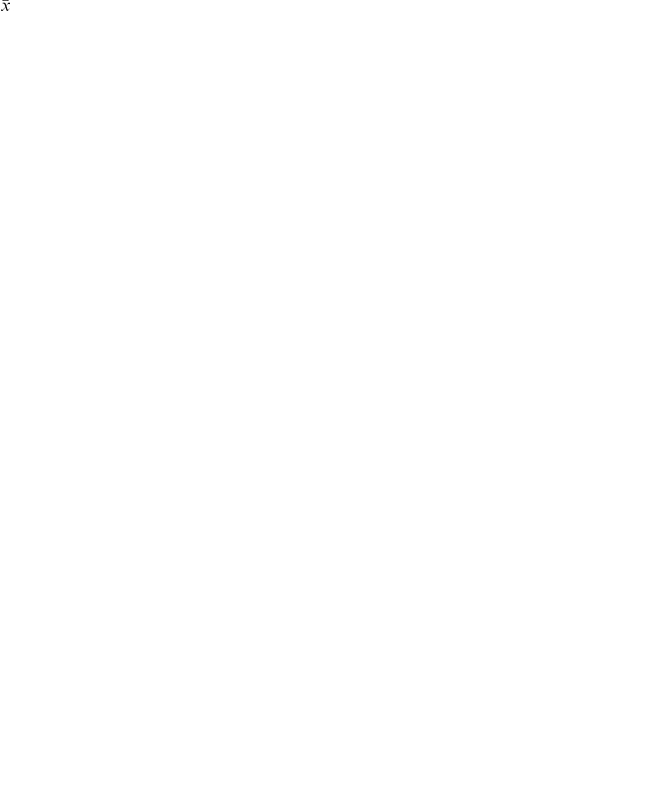
 and 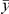 are the sample means of trait *x* and trait *y*, *s_x_* and *s_y_* are the sample standard deviations of trait *x* and trait *y* summed from i = 1 to n. Principal component (PC) analysis was performed to determine if the samples have different patterns of variation separating them into distinct groups and to provide loading coefficients for the PCs.

### Trait normality test, transformation and QTL detection

Testing for normal distribution of the traits, we employed the Anderson-Darling test, with the standard normal CDF Φ, which is calculated using:
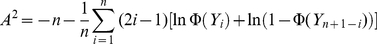
(2)where 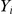 is the standardized value of variable 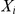, which is calculated as 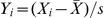, 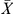 is the mean, and *s* is the standard deviation of each trait. Since we are dealing with large sample size, and adjusted 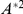 is employed in the test, which is calculated using:
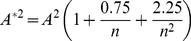
(3)


In this test, if *A* ^*2^ exceeds 0.75 then the hypothesis of normality is rejected at *p*≤0.05. For those traits that did not fit normal distribution, we used a BOXCOX function for normality transformation. The optimal λ value was derived by a SAS program. After BOXCOX transformation, the Anderson-Darling test was performed on the transformed data set. If the transformed data did not follow normal distribution, the traits were analyzed by a nonparametric mapping method employed in MapQTL5 [43]. As suggested by van Ooijen [43], we used a stringent significance level (p-value) for the individual nonparametric tests in order to obtain an overall significance under the assumption that the linkage group with a segregating QTL must reveal a gradient in the test statistic towards the locus with the closest linkage to the QTL. In this study, we report the marker with the highest K^*^ value at significance level above 0.005 to indicate the marker position that shows strongest linkage with the corresponding QTL.

For the traits following normal distribution or those that can be transformed into normal distribution, MapQTL 5.0 [43] was used to detect the underlying QTLs. We initially performed interval mapping to identify putative QTLs for these traits. Markers flanking a putative QTL were selected as cofactors and the selected marker was used as genetic background controls. When adding a new cofactor, if new peak appears, we employed additional cofactors until no further cofactor effect was found. Cofactors without an effect were deleted. The set of cofactors that were in use when the LOD profile stabilized was subsequently used in restricted MQM mapping [44]. LOD statistics were calculated at 1.0 cM intervals. Tests of 1000 genome-wide permutations were used to obtain an estimate of the number of type I errors (false positives). In the final MQM model the genetic effect and percentage of explained variance were estimated for each QTL and 2-LOD support intervals were established using restricted MQM mapping [45].

## Supporting Information

Table S1 Pair-wise correlation between all measured traits.
(0.09 MB XLS)

Table S2 Distribution test for the phenotypic data and parameters associated with each trait before and after BOXCOX transformation.
(0.02 MB DOC)

Table S3 Descriptive metrics for the detected QTLs underlying cell wall subcomponents in stem and root in Family 331.
(0.07 MB DOC)

Table S4 Distribution test of QTLs among chromosomes of Populus by Poisson calculator.
(0.02 MB DOC)

Table S5 Classification of the putative pleiotropic loci.
(0.04 MB DOC)
