# Supplementary material for: Differential Detection of Genetic Loci Underlying Stem and Root Lignin Content in Populus
Source: PLoS One. 2010 Nov 22;5(11):e14021. doi: 10.1371/journal.pone.0014021 (PMC2999904; doi:10.1371/journal.pone.0014021)
Supplement: Table S2 — Distribution test for the phenotypic data and parameters associated with each trait before and after BOXCOX transformation. (0.02 MB DOC) [file pone.0014021.s002.docx]

**Table S2. Distribution test for the phenotypic data and parameters associated with each trait before and after BOXCOX transformation.**

|  | **Original data** | | | | | **Box-Cox transformation** | | |
| --- | --- | --- | --- | --- | --- | --- | --- | --- |
| **Trait** | **Mean** | **Number of progeny** | **s.d.** | **A^2^*** | **Distribution before transformation** | **Highest lambda** | **A^2^*** | **Distribution after transformation** |
| **Lignin stem** | 24.28 | 292 | 1.059 | 1.585 | Non-normal | 3.44 | 0.596 | Normal |
| **Lignin root** | 22.25 | 284 | 1.868 | 4.499 | Non-normal | 4.59 | 0.539 | Normal |
| **SG ratio stem** | 2.010 | 288 | 0.1576 | 0.565 | Normal |  |  |  |
| **SG ration root** | 1.429 | 283 | 0.2219 | 3.496 | Non-normal | 2.07 | 0.745 | Normal |
| **Root m/z 41** | 0.6425 | 266 | 0.05416 | 2.137 | Non-normal | -0.8 | 1.033 | Non-normal |
| **Root m/z 43** | 4.933 | 266 | 0.4263 | 0.609 | Normal | -0.4 |  |  |
| **Root m/z 57** | 1.413 | 266 | 0.2351 | 4.945 | Non-normal | -1.2 | 0.706 | Normal |
| **Root m/z 58** | 0.7335 | 266 | 0.08279 | 0.428 | Normal |  |  |  |
| **Root m/z 60** | 1.697 | 266 | 0.547 | 17.359 | Non-normal | -1.7 | 0.146 | Normal |
| **Root m/z 73** | 1.941 | 266 | 0.5055 | 11.048 | Non-normal | -1.3 | 0.399 | Normal |
| **Root m/z 85** | 2.661 | 266 | 0.3001 | 0.711 | Normal |  |  |  |
| **Root m/z 94** | 0.6335 | 266 | 0.1638 | 1.488 | Non-normal | -0.1 | 0.413 | Normal |
| **Root m/z 97** | 1.191 | 266 | 0.2179 | 7.132 | Non-normal | -1.5 | 0.424 | Normal |
| **Root m/z 98** | 1.264 | 266 | 0.1413 | 9.706 | Non-normal | -4.2 | 0.657 | Normal |
| **Root m/z 114** | 1.375 | 266 | 0.2225 | 0.525 | Normal |  |  |  |
| **Root m/z 120** | 0.2552 | 266 | 0.02684 | 2.449 | Non-normal | 2.4 | 1.81 | Non-normal |
| **Root m/z 124** | 1.249 | 266 | 0.1858 | 1.349 | Non-normal | 2.2 | 0.193 | Normal |
| **Root m/z 126** | 1.358 | 266 | 0.09346 | 4.525 | Non-normal | -2.5 | 0.592 | Normal |
| **Root m/z 137** | 2.015 | 266 | 0.251 | 1.218 | Non-normal | 2.4 | 0.274 | Normal |
| **Root m/z 138** | 1.394 | 266 | 0.1753 | 0.501 | Normal |  |  |  |
| **Root m/z 144** | 0.3999 | 266 | 0.1024 | 9.709 | Non-normal | -1.2 | 0.699 | Normal |
| **Root m/z 150** | 0.6973 | 266 | 0.09036 | 0.232 | Normal |  |  |  |
| **Root m/z 154** | 0.9097 | 266 | 0.1237 | 0.398 | Normal |  |  |  |
| **Root m/z 164** | 0.5764 | 266 | 0.05759 | 0.743 | Normal |  |  |  |
| **Root m/z 167** | 1.609 | 266 | 0.1964 | 0.336 | Normal |  |  |  |
| **Root m/z 168** | 0.7644 | 266 | 0.0699 | 0.47 | Normal |  |  |  |
| **Root m/z 178** | 0.3971 | 266 | 0.03933 | 1.467 | Non-normal | 2.2 | 1.133 | Non-normal |
| **Root m/z 180** | 1.406 | 266 | 0.2368 | 1.874 | Non-normal | 2.2 | 0.223 | Normal |
| **Root m/z 182** | 0.5006 | 266 | 0.07566 | 1.368 | Non-normal | 1.2 | 1.126 | Non-normal |
| **Root m/z 194** | 0.4656 | 266 | 0.0735 | 2.047 | Non-normal | 2.4 | 0.559 | normal |
| **Root m/z 208** | 0.2294 | 266 | 0.04256 | 1.966 | Non-normal | 2 | 1.214 | Non-normal |
| **Root m/z 210** | 0.5368 | 266 | 0.1376 | 0.823 | Non-normal | 1.3 | 0.38 | Normal |
| **Stem m/z 41** | 0.2899 | 274 | 0.04447 | 2.958 | Non-normal | -0.4 | 1.409 | Non-normal |
| **Stem m/z 43** | 2.294 | 274 | 0.489 | 5.44 | Non-normal | -1.6 | 1.41 | Non-normal |
| **Stem m/z 57** | 1.3 | 274 | 0.1535 | 1.973 | Non-normal | -0.6 | 0.626 | Normal |
| **Stem m/z 58** | 0.733 | 274 | 0.132 | 4.837 | Non-normal | -1.3 | 1.237 | Non-normal |
| **Stem m/z 60** | 1.345 | 274 | 0.2388 | 2.208 | Non-normal | -0.6 | 0.201 | Normal |
| **Stem m/z 73** | 1.433 | 274 | 0.1868 | 1.209 | Non-normal | 0 | 0.421 | Normal |
| **Stem m/z 85** | 2.78 | 274 | 0.2971 | 0.978 | Non-normal | -0.6 | 0.385 | Normal |
| **Stem m/z 94** | 0.4229 | 274 | 0.08625 | 5.987 | Non-normal | -1.1 | 0.623 | Normal |
| **Stem m/z 97** | 1.236 | 274 | 0.14 | 1.961 | Non-normal | -0.9 | 0.371 | Normal |
| **Stem m/z 98** | 1.708 | 274 | 0.1784 | 2.921 | Non-normal | -1.8 | 0.415 | Normal |
| **Stem m/z 99** | 0.6379 | 274 | 0.06238 | 1.411 | Non-normal | -0.8 | 0.483 | Normal |
| **Stem m/z 114** | 1.438 | 274 | 0.2273 | 1.342 | Non-normal | -0.1 | 0.26 | Normal |
| **Stem m/z 120** | 0.1827 | 274 | 0.02421 | 3.184 | Non-normal | 0.2 | 3.144 | Non-normal |
| **Stem m/z 124** | 0.8718 | 274 | 0.08795 | 0.47 | Normal |  |  |  |
| **Stem m/z 126** | 1.238 | 274 | 0.1279 | 2.769 | Non-normal | -1.7 | 0.562 | Normal |
| **Stem m/z 137** | 1.456 | 274 | 0.2004 | 0.235 | Normal |  |  |  |
| **Stem m/z 138** | 0.8488 | 274 | 0.09978 | 3.403 | Non-normal | -1.5 | 0.459 | Normal |
| **Stem m/z 144** | 0.3492 | 274 | 0.05743 | 2.475 | Non-normal | -0.21 | 0.68 | Normal |
| **Stem m/z 150** | 0.7614 | 274 | 0.08851 | 1.359 | Non-normal | 0.3 | 1.149 | Non-normal |
| **Stem m/z 154** | 1.452 | 274 | 0.1558 | 0.282 | Normal |  |  |  |
| **Stem m/z 164** | 0.5492 | 274 | 0.05649 | 0.574 | Normal |  |  |  |
| **Stem m/z 167** | 1.869 | 274 | 0.3508 | 1.997 | Non-normal | 1.8 | 1.023 | Non-normal |
| **Stem m/z 168** | 0.8764 | 274 | 0.1143 | 0.926 | Non-normal | 1.8 | 0.509 | Normal |
| **Stem m/z 178** | 0.4727 | 274 | 0.04763 | 0.89 | Non-normal | 1.8 | 0.737 | Normal |
| **Stem m/z 180** | 2.124 | 274 | 0.2801 | 1.112 | Non-normal | 2 | 0.392 | Normal |
| **Stem m/z 182** | 0.8927 | 274 | 0.1265 | 0.923 | Non-normal | 1.9 | 0.406 | Normal |
| **Stem m/z 194** | 0.7749 | 274 | 0.1168 | 1.626 | Non-normal | 2.1 | 0.575 | Normal |
| **Stem m/z 208** | 0.4654 | 274 | 0.06729 | 1.421 | Non-normal | 2.3 | 0.507 | Normal |
| **Stem m/z 210** | 1.578 | 274 | 0.2714 | 0.794 | Non-normal | 1.7 | 0.253 | Normal |

Critical value = 0.752 at 5% level.
