# Supplementary material for: Differential Detection of Genetic Loci Underlying Stem and Root Lignin Content in Populus
Source: PLoS One. 2010 Nov 22;5(11):e14021. doi: 10.1371/journal.pone.0014021 (PMC2999904; doi:10.1371/journal.pone.0014021)
Supplement: Table S3 — Descriptive metrics for the detected QTLs underlying cell wall subcomponents in stem and root in Family 331. (0.07 MB DOC) [file pone.0014021.s003.docx]

**Table S3. Descriptive metrics for the detected QTLs underlying cell wall subcomponents in stem and root in Family 331.**

M, maternal parent; P, Paternal parent.

The physical position of each QTL on the *Populus* genome is depicted in Figure 2.

| **Tissue/ Trait** | **Linkage Group** | **LOD Peak Position (cM)** | **Peak LOD** | **LOD** | **2-LOD** | **% Phenotypic Variance Explained** | **Origin of the largest effect allele(s)** | **Mean value** | | | |
| --- | --- | --- | --- | --- | --- | --- | --- | --- | --- | --- | --- |
|  |  |  |  | **Threshold** | **Interval (cM)** |  |  | **ac** | **ad** | **bc** | **bd** |
| **Root** |  |  |  |  |  |  |  |  |  |  |  |
| **lignin** | VI | 66.679 | 5.06 | 2.46 | 61.067-71.158 | 8.9 | M | 63.980 | 61.421 | 59.379 | 48.092 |
|  | X | 67.724 | 2.91 | 1.88 | 63.867-70.043 | 5.2 | M | 56.537 | 53.570 | 59.121 | 68.427 |
|  | X | 130.174 | 3.81 | 2.01 | 90.634-130.174 | 11.1 | M | 47.522 | 56.504 | 59.663 | 65.781 |
|  | XIV | 5.661 | 2.71 | 1.75 | 0.000-9.353 | 8.4 | M | 51.738 | 61.204 | 66.180 | 53.340 |
| **SG** | III | 57.823 | 5.19 | 2.74 | 48.823-65.823 | 14.9 | P | 2.786 | 3.030 | 2.723 | 3.889 |
|  | VI | 71.158 | 2.82 | 2.35 | 62.624-71.158 | 5.4 | P | 2.864 | 3.226 | 3.429 | 2.757 |
|  | X | 48.508 | 3.19 | 1.97 | 47.244-52.508 | 6.0 | M | 3.515 | 3.054 | 2.867 | 2.724 |
|  | XIII | 50.389 | 3.21 | 2.11 | 39.936-60.298 | 6.6 | M | 3.160 | 3.117 | 2.569 | 3.448 |
| **m/z 43** | II | 38.205 | 2.51 | 2.32 | 25.530-43.205 | 11.0 | M | 5.140 | 4.947 | 4.739 | 4.901 |
|  | II | 136.839 | 6.78 | 3.01 | 129.601-140.839 | 22.9 | P | 4.828 | 5.450 | 4.813 | 4.931 |
|  | IX | 31.652 | 2.84 | 2.22 | 24.107-31.652 | 4.8 | P | 4.961 | 4.855 | 4.845 | 5.066 |
|  | XIV | 3.000 | 2.74 | 2.34 | 0.000-21.373 | 5.6 | M,P | 5.090 | 4.895 | 4.895 | 4.829 |
| **m/z 57** | I | 158.274 | 2.63 | 2.49 | 151.643-158.274 | 5.1 | M | 0.636 | 0.700 | 0.705 | 0.679 |
|  | VI | 64.624 | 2.83 | 2.08 | 52.986-71.002 | 6.9 | M,P | 0.680 | 0.697 | 0.716 | 0.633 |
| **m/z 58** | II | 132.839 | 4.02 | 2.37 | 57.130-66.785 | 8.9 | P | 0.710 | 0.796 | 0.736 | 0.726 |
|  | II | 62.130 | 3.11 | 2.87 | 127.601-139.839 | 12.6 | P | 0.776 | 0.695 | 0.725 | 0.733 |
|  | X | 21.898 | 2.54 | 2.06 | 16.826-27.790 | 8.8 | M,P | 0.767 | 0.712 | 0.712 | 0.745 |
|  | X | 106.045 | 3.29 | 2.24 | 92.634-130.174 | 6.3 | M,P | 0.766 | 0.729 | 0.730 | 0.705 |
|  | XI | 18.680 | 2.79 | 1.83 | 16.289-32.650 | 5.0 | M,P | 0.716 | 0.751 | 0.752 | 0.714 |
|  | XII | 15.402 | 2.62 | 1.92 | 15.402-19.787 | 4.5 | M,P | 0.758 | 0.722 | 0.742 | 0.713 |
|  | XIV | 6.661 | 3.87 | 2.39 | 1.000-9.353 | 10.8 | P | 0.755 | 0.711 | 0.701 | 0.761 |
| **m/z 60** | VI | 66.679 | 4.01 | 2.51 | 61.067-71.158 | 7.1 | M,P | 0.502 | 0.485 | 0.495 | 0.398 |
|  | VIII | 17.566 | 2.92 | 2.00 | 16.372-19.566 | 5.5 | M,P | 0.478 | 0.511 | 0.460 | 0.402 |
|  | X | 104.634 | 4.26 | 2.41 | 91.634-112.926 | 8.7 | M | 0.396 | 0.474 | 0.476 | 0.535 |
|  | XIV | 4.661 | 3.65 | 1.85 | 1.000-8.353 | 11.5 | M | 0.404 | 0.499 | 0.543 | 0.434 |
| **m/z 73** | VI | 66.679 | 4.33 | 2.64 | 61.067-71.158 | 7.6 | M,P | 0.483 | 0.471 | 0.468 | 0.402 |
|  | VIII | 17.566 | 2.84 | 2.13 | 16.372-19.566 | 5.2 | M,P | 0.464 | 0.487 | 0.444 | 0.412 |
|  | X | 103.634 | 4.36 | 2.50 | 90.634-130.174 | 9.0 | P | 0.405 | 0.457 | 0.456 | 0.509 |
|  | XIV | 5.661 | 3.07 | 1.92 | 0.000-8.353 | 9.4 | M | 0.413 | 0.463 | 0.507 | 0.433 |
| **m/z 85** | I | 22.804 | 2.93 | 1.84 | 8.598-22.804 | 5.4 | M | 2.567 | 2.644 | 2.661 | 2.763 |
|  | VI | 70.408 | 3.88 | 2.30 | 52.986-71.798 | 6.7 | M | 2.539 | 2.650 | 2.675 | 2.766 |
|  | X | 67.724 | 3.68 | 2.14 | 61.005-71.043 | 6.4 | M,P | 2.686 | 2.713 | 2.666 | 2.473 |
|  | X | 100.634 | 3.49 | 2.45 | 84.634-124.515 | 7.8 | M,P | 2.754 | 2.665 | 2.683 | 2.509 |
|  | XIII | 38.936 | 2.65 | 1.86 | 26.725-44.389 | 4.8 | P | 2.707 | 2.614 | 2.583 | 2.749 |
|  | XIV | 5.661 | 2.68 | 1.93 | 0.000-8.353 | 7.8 | P | 2.703 | 2.638 | 2.536 | 2.744 |
| **m/z 94** | X | 66.662 | 3.01 | 2.00 | 54.637-68.724 | 5.8 | M,P | 1.054 | 1.056 | 1.047 | 1.037 |
|  | X | 103.634 | 4.11 | 2.44 | 91.634-117.515 | 8.4 | M,P | 1.062 | 1.051 | 1.048 | 1.039 |
|  | XVI | 49.868 | 2.50 | 1.98 | 48.008-60.868 | 7.7 | P | 1.043 | 1.059 | 1.055 | 1.043 |
|  | XVIII | 42.692 | 3.39 | 2.31 | 41.923-45.260 | 17.3 | M,P | 1.070 | 1.058 | 1.039 | 1.048 |
| **m/z 97** | VI | 65.624 | 4.24 | 2.46 | 60.067-71.158 | 8.1 | M,P | 0.872 | 0.835 | 0.824 | 0.726 |
|  | VIII | 17.566 | 2.80 | 2.01 | 16.372-19.568 | 5.2 | M,P | 0.823 | 0.865 | 0.795 | 0.743 |
|  | X | 67.724 | 3.63 | 2.52 | 63.867-71.834 | 6.3 | M | 0.789 | 0.778 | 0.815 | 0.928 |
|  | X | 101.634 | 4.74 | 3.01 | 89.634-127.515 | 10.1 | P | 0.728 | 0.816 | 0.810 | 0.908 |
| **m/z 98** | XIV | 4.661 | 3.03 | 2.11 | 0.000-8.353 | 9.1 | M | 0.758 | 0.807 | 0.902 | 0.775 |
|  | VI | 71.158 | 4.86 | 2.62 | 64.624-71.798 | 11.3 | M,P | 0.500 | 0.412 | 0.392 | 0.366 |
|  | VIII | 9.784 | 2.51 | 2.03 | 3.729-15.561 | 7.0 | M,P | 0.488 | 0.398 | 0.395 | 0.390 |
|  | X | 67.724 | 2.88 | 1.99 | 64.867-71.834 | 5.2 | M | 0.415 | 0.384 | 0.411 | 0.491 |
|  | X | 130.174 | 3.66 | 2.31 | 92.634-130.174 | 9.5 | M | 0.354 | 0.397 | 0.420 | 0.471 |
|  | XIII | 35.943 | 2.62 | 2.02 | 25.725-50.389 | 5.6 | M,P | 0.381 | 0.447 | 0.448 | 0.377 |
| **m/z 114** | II | 62.130 | 3.00 | 2.14 | 56.130-64.785 | 18.8 | M,P | 1.469 | 1.220 | 1.441 | 1.364 |
|  | VI | 63.624 | 3.80 | 2.43 | 51.986-71.158 | 8.0 | M | 1.312 | 1.343 | 1.355 | 1.477 |
|  | VIII | 10.097 | 2.58 | 1.98 | 3.729-13.035 | 5.0 | M | 1.333 | 1.314 | 1.434 | 1.405 |
|  | X | 67.724 | 4.34 | 2.48 | 60.005-68.724 | 7.5 | M,P | 1.417 | 1.414 | 1.352 | 1.235 |
|  | X | 106.045 | 4.92 | 2.56 | 91.634-130.174 | 9.2 | M,P | 1.477 | 1.363 | 1.364 | 1.281 |
|  | XIV | 6.661 | 4.52 | 2.47 | 4.661-8.353 | 12.3 | P | 1.446 | 1.334 | 1.267 | 1.444 |
|  | XVI | 49.868 | 2.64 | 2.08 | 48.008-61.868 | 8.9 | M,P | 1.313 | 1.442 | 1.436 | 1.301 |
| **m/z 124** | VI | 69.685 | 3.67 | 2.17 | 49.986-71.158 | 6.5 | M,P | 1.827 | 1.704 | 1.736 | 1.467 |
|  | VIII | 13.035 | 3.11 | 2.36 | 12.035-18.566 | 8.8 | M,P | 1.687 | 1.830 | 1.731 | 1.417 |
|  | X | 130.174 | 3.08 | 2.65 | 91.634-130.174 | 8.3 | M | 1.458 | 1.665 | 1.696 | 1.856 |
| **m/z 126** | II | 112.601 | 3.01 | 2.11 | 97.932-123.601 | 6.0 | M,P | 0.499 | 0.470 | 0.484 | 0.458 |
|  | XII | 19.787 | 2.62 | 2.05 | 15.042-29.945 | 5.0 | M | 0.461 | 0.465 | 0.493 | 0.489 |
|  | XIV | 1.000 | 3.06 | 2.44 | 0.000-23.169 | 6.2 | M | 0.457 | 0.465 | 0.488 | 0.495 |
| **m/z 137** | VI | 71.158 | 3.07 | 2.31 | 48.986-71.798 | 8.3 | M,P | 6.191 | 5.322 | 5.674 | 4.948 |
|  | VIII | 14.035 | 2.75 | 2.12 | 11.035-18.586 | 7.7 | M,P | 5.623 | 5.852 | 5.697 | 4.719 |
|  | X | 130.174 | 3.14 | 2.54 | 91.634-130.174 | 8.1 | M | 4.934 | 5.299 | 5.576 | 6.084 |
| **m/z 138** | II | 46.319 | 3.53 | 2.32 | 39.071-63.785 | 13.9 | P | 1.341 | 1.374 | 1.352 | 1.504 |
|  | II | 110.373 | 2.85 | 2.15 | 100.006-127.601 | 5.0 | M | 1.382 | 1.355 | 1.464 | 1.383 |
|  | III | 75.932 | 2.99 | 2.10 | 68.928-84.166 | 7.5 | M,P | 1.434 | 1.441 | 1.348 | 1.332 |
|  | X | 66.444 | 2.50 | 1.87 | 55.637-71.043 | 4.9 | M,P | 1.393 | 1.370 | 1.451 | 1.335 |
|  | XV | 78.154 | 2.51 | 1.96 | 59.894-87.344 | 7.6 | M,P | 1.478 | 1.365 | 1.388 | 1.344 |
|  | XVIII | 11.000 | 3.18 | 2.11 | 0.000-41.923 | 10.6 | P | 1.317 | 1.414 | 1.395 | 1.477 |
| **m/z 144** | VI | 65.624 | 4.09 | 2.74 | 57.032-71.158 | 8.0 | M,P | 3.410 | 3.312 | 3.351 | 2.845 |
|  | VIII | 17.566 | 2.61 | 1.95 | 7.784-18.566 | 4.9 | M,P | 3.263 | 3.434 | 3.167 | 2.923 |
|  | X | 67.724 | 3.39 | 1.99 | 61.005-71.834 | 5.9 | M | 3.136 | 3.098 | 3.202 | 3.707 |
|  | X | 101.634 | 3.41 | 2.38 | 85.634-130.174 | 7.4 | P | 2.916 | 3.232 | 3.219 | 3.568 |
|  | XIV | 4.661 | 3.67 | 2.36 | 0.000-7.353 | 10.9 | M | 2.986 | 3.163 | 3.646 | 3.056 |
| **m/z 150** | VI | 64.624 | 4.83 | 2.87 | 51.986-71.158 | 9.9 | M,P | 0.728 | 0.700 | 0.715 | 0.653 |
|  | VIII | 13.035 | 3.40 | 2.44 | 11.035-17.566 | 10.1 | M,P | 0.691 | 0.725 | 0.712 | 0.648 |
|  | X | 106.045 | 3.11 | 2.41 | 90.634-130.174 | 5.9 | M | 0.667 | 0.695 | 0.700 | 0.732 |
| **m/z 154** | VI | 65.624 | 5.12 | 3.02 | 60.067-71.158 | 10.0 | M,P | 0.925 | 0.927 | 0.946 | 0.846 |
|  | VII | 8.615 | 2.82 | 2.11 | 1.615-41.263 | 15.4 | P | 0.973 | 0.927 | 0.829 | 0.895 |
|  | VIII | 7.784 | 3.33 | 2.30 | 3.729-10.097 | 7.7 | M,P | 0.959 | 0.930 | 0.896 | 0.866 |
|  | XIV | 5.661 | 3.94 | 2.44 | 0.000-8.353 | 11.1 | M | 0.870 | 0.938 | 0.964 | 0.875 |
| **m/z 164** | IV | 33.315 | 2.86 | 2.05 | 8.315-46.064 | 5.7 | M,P | 0.570 | 0.570 | 0.599 | 0.565 |
|  | V | 121.385 | 3.17 | 2.07 | 117.972-121.385 | 5.8 | P | 0.566 | 0.575 | 0.561 | 0.597 |
|  | VI | 61.067 | 3.50 | 2.41 | 45.140-69.685 | 7.4 | M,P | 0.591 | 0.576 | 0.589 | 0.551 |
| **m/z 167** | III | 57.823 | 2.73 | 2.03 | 48.687-68.100 | 8.6 | P | 1.562 | 1.637 | 1.555 | 1.699 |
|  | VII | 38.263 | 3.01 | 2.44 | 1.615-59.435 | 6.8 | P | 1.652 | 1.642 | 1.507 | 1.603 |
|  | X | 49.508 | 3.03 | 2.11 | 47.244-54.508 | 5.8 | M,P | 1.682 | 1.581 | 1.566 | 1.609 |
| **m/z 168** | VII | 46.148 | 4.97 | 2.41 | 39.263-52.238 | 8.4 | P | 0.765 | 0.790 | 0.730 | 0.763 |
|  | XII | 39.610 | 3.05 | 2.31 | 24.531-46.056 | 5.7 | P | 0.736 | 0.779 | 0.763 | 0.776 |
| **m/z 180** | VI | 66.679 | 3.27 | 2.12 | 60.067-71.158 | 5.9 | M,P | 2.358 | 2.304 | 2.253 | 1.905 |
|  | VIII | 17.566 | 2.98 | 2.01 | 6.198-18.566 | 5.7 | M,P | 2.325 | 2.355 | 2.170 | 1.868 |
|  | X | 130.174 | 3.87 | 2.53 | 91.634-130.174 | 12.8 | M | 1.757 | 2.218 | 2.279 | 2.466 |
|  | XIV | 3.000 | 2.93 | 2.34 | 0.000-7.353 | 9.0 | M | 1.922 | 2.350 | 2.489 | 2.061 |
| **m/z 194** | I | 158.274 | 3.06 | 2.31 | 152.643-158.274 | 6.1 | M | 0.144 | 0.178 | 0.179 | 0.161 |
|  | XII | 15.402 | 2.53 | 1.98 | 15.402-18.793 | 4.4 | P | 0.154 | 0.177 | 0.154 | 0.180 |
|  | XIV | 3.000 | 2.75 | 1.87 | 0.000-7.353 | 8.8 | P | 0.152 | 0.189 | 0.184 | 0.151 |
|  | XVI | 36.989 | 2.71 | 2.21 | 32.823-41.989 | 9.4 | M | 0.138 | 0.171 | 0.185 | 0.172 |
| **m/z 210** | No QTL |  |  |  |  |  |  |  |  |  |  |
| **Stem** |  |  |  |  |  |  |  |  |  |  |  |
| **lignin** | II | 175.443 | 2.85 | 1.91 | 167.589-175.443 | 6.9 | M | 76.790 | 65.911 | 73.040 | 72.594 |
|  | VI | 71.158 | 4.29 | 2.33 | 60.067-71.108 | 7.6 | P | 77.298 | 73.237 | 72.024 | 65.473 |
|  | VI | 82.470 | 4.21 | 2.05 | 78.800-105.578 | 7.3 | P | 78.884 | 72.098 | 70.951 | 67.513 |
|  | X | 80.148 | 2.71 | 2.41 | 75.202-105.634 | 4.6 | M | 66.996 | 73.039 | 74.073 | 75.285 |
| **SG** | II | 90.759 | 5.51 | 2.44 | 88.759-92.085 | 12.5 | M | 1.934 | 2.032 | 2.096 | 1.994 |
|  | III | 23.623 | 3.22 | 2.35 | 10.724-32.197 | 6.4 | P | 1.986 | 2.010 | 1.970 | 2.079 |
|  | VI | 80.744 | 2.58 | 1.86 | 71.158-85.787 | 5.2 | P | 2.045 | 1.969 | 2.044 | 1.974 |
|  | VIII | 25.736 | 2.69 | 2.21 | 10.097-27.222 | 5.2 | P | 2.011 | 2.042 | 1.942 | 2.027 |
|  | XIV | 50.366 | 3.31 | 2.40 | 40.764-57.363 | 6.7 | M | 2.021 | 2.056 | 2.009 | 1.939 |
| **m/z 57** | VI | 72.798 | 2.57 | 2.03 | 71.158-87.787 | 5.7 | P | 0.859 | 0.877 | 0.839 | 0.866 |
|  | VII | 95.905 | 2.51 | 2.41 | 79.691-107.345 | 5.4 | M,P | 0.872 | 0.874 | 0.857 | 0.840 |
| **m/z 60** | II | 175.443 | 2.50 | 2.12 | 163.589-175.443 | 5.7 | M,P | 0.876 | 0.816 | 0.853 | 0.852 |
|  | VI | 68.679 | 2.55 | 1.86 | 62.624-71.158 | 4.3 | P | 0.862 | 0.869 | 0.825 | 0.842 |
| **m/z 73** | VI | 68.685 | 5.14 | 2.99 | 63.624-71.158 | 8.4 | M | 0.300 | 0.329 | 0.369 | 0.402 |
|  | VI | 82.470 | 5.09 | 2.87 | 77.800-95.530 | 8.9 | M | 0.293 | 0.328 | 0.372 | 0.398 |
|  | X | 94.634 | 2.78 | 2.36 | 78.471-125.515 | 6.5 | M,P | 0.390 | 0.360 | 0.346 | 0.294 |
| **m/z 85** | VI | 71.158 | 3.89 | 2.33 | 65.624-74.024 | 8.1 | P | 0.546 | 0.559 | 0.532 | 0.542 |
|  | VII | 101.905 | 2.51 | 2.21 | 82.691-107.345 | 6.8 | M,P | 0.545 | 0.556 | 0.547 | 0.532 |
| **m/z 94** | II | 121.601 | 3.03 | 2.23 | 94.389-153.000 | 7.5 | P | 2.825 | 2.894 | 2.515 | 2.617 |
|  | IV | 104.779 | 2.56 | 2.01 | 93.041-119.250 | 5.7 | M,P | 3.050 | 2.680 | 2.634 | 2.622 |
|  | X | 66.662 | 3.28 | 2.12 | 57.005-70.043 | 6.1 | P | 2.801 | 2.794 | 2.514 | 2.545 |
|  | X | 100.634 | 5.50 | 2.77 | 89.634-123.515 | 12.3 | M,P | 2.890 | 2.821 | 2.510 | 2.445 |
|  | XIV | 32.724 | 2.69 | 1.85 | 10.101-61.684 | 9.4 | P | 2.558 | 2.881 | 2.500 | 2.815 |
| **m/z 97** | IV | 115.229 | 3.11 | 2.01 | 104.996-119.250 | 7.6 | P | 0.810 | 0.848 | 0.798 | 0.853 |
|  | VI | 68.679 | 5.11 | 3.01 | 61.624-71.158 | 8.3 | M,P | 0.867 | 0.847 | 0.829 | 0.800 |
|  | VI | 87.787 | 4.42 | 2.22 | 78.800-93.562 | 10.3 | M,P | 0.881 | 0.839 | 0.812 | 0.816 |
|  | VIII | 47.512 | 2.89 | 2.31 | 46.325-67.826 | 6.4 | P | 0.831 | 0.871 | 0.819 | 0.820 |
|  | X | 115.515 | 3.00 | 2.41 | 85.634-127.515 | 7.7 | M | 0.830 | 0.809 | 0.833 | 0.872 |
|  | XVI | 49.868 | 2.91 | 2.02 | 48.008-69.220 | 9.0 | M | 0.801 | 0.822 | 0.868 | 0.847 |
| **m/z 98** | VI | 73.024 | 2.76 | 1.82 | 61.624-88.787 | 6.2 | P | 0.407 | 0.413 | 0.374 | 0.379 |
| **m/z 99** | IV | 113.229 | 2.87 | 1.97 | 102.779-119.250 | 7.1 | P | 1.398 | 1.438 | 1.411 | 1.476 |
|  | X | 76.471 | 3.22 | 2.24 | 72.202-86.634 | 8.5 | P | 1.399 | 1.480 | 1.456 | 1.436 |
|  | XVI | 49.868 | 2.75 | 2.19 | 48.008-69.220 | 8.8 | M | 1.389 | 1.437 | 1.478 | 1.462 |
| **m/z 114** | II | 169.589 | 2.50 | 2.44 | 143.313-175.443 | 4.3 | M,P | 0.969 | 0.961 | 0.968 | 0.964 |
|  | VI | 87.787 | 2.66 | 2.19 | 64.624-87.787 | 6.2 | P | 0.969 | 0.969 | 0.960 | 0.965 |
|  | X | 107.926 | 3.68 | 2.68 | 89.634-119.515 | 6.1 | M | 0.963 | 0.964 | 0.964 | 0.973 |
| **m/z 124** | V | 63.329 | 2.79 | 2.46 | 55.823-72.525 | 5.1 | M,P | 0.898 | 0.871 | 0.854 | 0.847 |
|  | VI | 79.800 | 5.06 | 3.45 | 71-798-91.775 | 9.8 | P | 0.904 | 0.898 | 0.856 | 0.838 |
|  | VI | 107.578 | 4.79 | 2.24 | 101.578-141.324 | 13.8 | M,P | 0.930 | 0.873 | 0.846 | 0.852 |
|  | XII | 37.610 | 3.60 | 2.31 | 26.945-46.056 | 6.4 | M,P | 0.898 | 0.875 | 0.878 | 0.836 |
|  | XVIII | 39.451 | 2.64 | 2.05 | 18.000-45.260 | 6.0 | P | 0.839 | 0.899 | 0.866 | 0.876 |
| **m/z 126** | II | 170.636 | 2.60 | 2.26 | 164.589-175.443 | 4.3 | M,P | 1.501 | 1.385 | 1.471 | 1.466 |
|  | V | 64.857 | 3.22 | 2.76 | 54.823-72.525 | 5.6 | M,P | 1.518 | 1.452 | 1.402 | 1.429 |
|  | VI | 72.798 | 3.26 | 2.28 | 63.624-95.903 | 6.7 | P | 1.470 | 1.531 | 1.391 | 1.442 |
|  | VII | 94.905 | 2.67 | 1.89 | 80.691-107.345 | 5.2 | P | 1.472 | 1.523 | 1.436 | 1.402 |
|  | XIV | 14.232 | 2.53 | 1.92 | 8.353-22.806 | 5.1 | M | 1.443 | 1.418 | 1.422 | 1.523 |
| **m/z 138** | II | 168.589 | 2.72 | 1.87 | 151.045-175.443 | 5.1 | P | 1.286 | 1.393 | 1.267 | 1.294 |
|  | V | 65.661 | 2.73 | 2.34 | 62.334-73.051 | 4.9 | P | 1.245 | 1.318 | 1.353 | 1.360 |
|  | VIII | 4.737 | 3.13 | 2.45 | 0.000-28.282 | 13.4 | P | 1.174 | 1.394 | 1.315 | 1.346 |
|  | X | 107.045 | 6.83 | 3.42 | 56.637-70.043 | 11.5 | M,P | 1.373 | 1.379 | 1.248 | 1.213 |
|  | X | 67.724 | 3.47 | 2.21 | 94.634-123.515 | 6.1 | M,P | 1.376 | 1.326 | 1.238 | 1.267 |
| **m/z 144** | VI | 102.578 | 3.06 | 2.14 | 83.470-114.578 | 6.4 | M,P | 1.267 | 1.257 | 1.251 | 1.238 |
|  | X | 107.926 | 2.50 | 2.33 | 81.148-121.515 | 4.3 | M | 1.242 | 1.249 | 1.253 | 1.266 |
|  | VI | 41.576 | 2.69 | 2.25 | 35.576-45.140 | 7.3 | M | 1.242 | 1.258 | 1.272 | 1.247 |
| **m/z 154** | XV | 22.119 | 2.93 | 2.24 | 15.000-26.119 | 17.0 | M | 1.498 | 1.367 | 1.433 | 1.539 |
| **m/z 164** | XIV | 28.806 | 4.97 | 2.24 | 24.806-37.724 | 17.2 | P | 0.542 | 0.542 | 0.524 | 0.585 |
|  | XIV | 13.232 | 4.70 | 2.21 | 8.353-21.325 | 9.1 | P | 0.537 | 0.542 | 0.536 | 0.575 |
| **m/z 168** | No QTL |  |  |  |  |  |  |  |  |  |  |
| **m/z 178** | III | 38.687 | 3.04 | 2.45 | 30.197-44.687 | 5.2 | M,P | 0.269 | 0.260 | 0.269 | 0.244 |
|  | VII | 91.309 | 3.14 | 2.84 | 81.691-106.905 | 5.5 | P | 0.265 | 0.276 | 0.253 | 0.251 |
|  | VIII | 13.035 | 2.55 | 2.10 | 9.784-18.566 | 5.8 | M,P | 0.269 | 0.252 | 0.273 | 0.248 |
|  | XV | 85.176 | 3.03 | 2.31 | 76.154-86.176 | 13.8 | P | 0.237 | 0.282 | 0.271 | 0.249 |
| **m/z 180** | II | 170.636 | 3.20 | 2.44 | 167.589-175.443 | 5.3 | M,P | 4.889 | 4.143 | 4.677 | 4.656 |
|  | VI | 68.679 | 3.41 | 2.76 | 62.624-95.093 | 5.6 | P | 4.855 | 4.870 | 4.242 | 4.424 |
|  | VII | 91.309 | 2.81 | 2.10 | 82.691-106.905 | 4.8 | P | 4.601 | 4.984 | 4.534 | 4.291 |
| **m/z 182** | II | 115.601 | 2.88 | 1.99 | 98.006-129.601 | 5.9 | M,P | 0.897 | 0.785 | 0.841 | 0.766 |
| **m/z 194** | No QTL |  |  |  |  |  |  |  |  |  |  |
| **m/z 208** | XIV | 32.724 | 2.65 | 2.11 | 15.232-40.724 | 10.3 | P | 0.187 | 0.197 | 0.151 | 0.177 |
| **m/z 210** | No QTL |  |  |  |  |  |  |  |  |  |  |
