# Supplementary material for: Differential Detection of Genetic Loci Underlying Stem and Root Lignin Content in Populus
Source: PLoS One. 2010 Nov 22;5(11):e14021. doi: 10.1371/journal.pone.0014021 (PMC2999904; doi:10.1371/journal.pone.0014021)
Supplement: Table S4 — Distribution test of QTLs among chromosomes of Populus by Poisson calculator. (0.02 MB DOC) [file pone.0014021.s004.docx]

**Table S4. Distribution test of QTLs among chromosomes of *Populus* by Poisson calculator.**

| **Chromosome** | **Physical length (bp)** | **Observed No. of QTLs** | **Expect No. of QTLs** | **Distribution test (p value)** | **Significance** $P(m_{ij}<\lambda ij)$ or $P(m_{ij}>\lambda ij)$ |
| --- | --- | --- | --- | --- | --- |
| I | 35571569 | 4 | 21 | 0.0000 | **- |
| II | 24482572 | 18 | 14 | 0.0592 |  |
| III | 19129466 | 5 | 11 | 0.0210 | *- |
| IV | 16625654 | 4 | 10 | 0.0230 | *- |
| V | 17991592 | 4 | 10 | 0.0143 | *- |
| VI | 18519121 | 35 | 11 | 0.0000 | **+ |
| VII | 12805987 | 10 | 7 | 0.0843 |  |
| VIII | 16228216 | 17 | 9 | 0.0084 | **+ |
| IX | 12523952 | 1 | 7 | 0.0050 | **- |
| X | 21101489 | 37 | 12 | 0.0000 | **+ |
| XI | 15120528 | 2 | 9 | 0.0059 | **- |
| XII | 14142880 | 5 | 8 | 0.0841 |  |
| XIII | 13101108 | 3 | 8 | 0.0362 | *- |
| XIV | 14699529 | 20 | 9 | 0.0003 | **+ |
| XV | 10599685 | 5 | 6 | 0.1560 |  |
| XVI | 13661513 | 6 | 8 | 0.1238 |  |
| XVII | 6060117 | 0 | 4 | 0.0295 | **- |
| XVIII | 13470992 | 3 | 8 | 0.0318 | *- |
| XIX | 12003701 | 0 | 7 | 0.0009 | **- |

Note: * indicates significance at α < 0.05; ** at α < 0.01; “-” following the “*” indicates less than expected number at corresponding significant level; “+” following the “*” indicates more abundant than expected number at corresponding significant level.
