# Supplementary material for: Differential Detection of Genetic Loci Underlying Stem and Root Lignin Content in Populus
Source: PLoS One. 2010 Nov 22;5(11):e14021. doi: 10.1371/journal.pone.0014021 (PMC2999904; doi:10.1371/journal.pone.0014021)
Supplement: Table S5 — Classification of the putative pleiotropic loci. (0.04 MB DOC) [file pone.0014021.s005.docx]

**Table S5. Classification of the putative pleiotropic loci and differential QTL detection in stems and roots.**

The numbers in the “pleiotropic loci” column represent the arbitrary sequential designation of each pleiotropic locus.

| **Trait** | **Chromosome** | **LOD Peak Position** | **Pleiotropic Loci** | **Tissue Specificity** | **Pleiotropic Interval** | **Correlation Group** |
| --- | --- | --- | --- | --- | --- | --- |
| Stem m/z 58 | I | 8.59 |  |  |  | III |
| Root m/z 85 | I | 21.80 |  |  |  | IV |
| Root m/z 184 | I | 158.26 | 1 | Root specific | 0.00 | II |
| Root m/z 57 |  | 158.26 |  |  |  | IV |
| Root m/z 43 | II | 38.19 | 2 | Root specific | 8.11 | IV |
| Root m/z 138 |  | 46.32 |  |  |  | II |
| Root m/z 114 | II | 62.13 | 3 | Root specific | 0.00 | IV |
| Root m/z 58 |  | 62.13 |  |  |  | IV |
| Root m/z 168 | II | 78.17 |  |  |  | II |
| Stem SG | II | 90.76 |  |  |  | I |
| Root m/z 138 | II | 110.37 | 4 | Root and stem | 10.23 | II |
| Root m/z 125 |  | 112.60 |  |  |  | IV |
| Stem m/z 172 |  | 115.60 |  |  |  | I |
| Stem m/z 94 |  | 120.60 |  |  |  | III |
| Root m/z 58 | II | 132.84 | 5 | Root specific | 4.00 | IV |
| Root m/z 43 |  | 136.84 |  |  |  | IV |
| Stem m/z 138 | II | 158.59 | 6 | Stem specific | 6.85 | I |
| Stem m/z 114 |  | 159.59 |  |  |  | III |
| Stem m/z 125 |  | 160.64 |  |  |  | III |
| Stem m/z 170 |  | 160.64 |  |  |  | I |
| Stem lignin |  | 165.44 |  |  |  | I |
| Stem m/z 60 |  | 165.44 |  |  |  | III |
| Stem SG | III | 22.62 |  |  |  | I |
| Stem m/z 168 | III | 38.69 |  |  |  | I |
| Root SG | III | 57.82 | 7 | Root specific | 0.00 | II |
| Root m/z 157 |  | 57.82 |  |  |  | II |
| Root m/z 138 | III | 75.93 |  |  |  | II |
| Root m/z 154 | IV | 33.32 |  |  |  | II |
| Stem m/z 94 | IV | 104.78 | 8 | Stem specific | 10.44 | III |
| Stem m/z 99 |  | 113.22 |  |  |  | III |
| Stem m/z 97 |  | 115.22 |  |  |  | III |
| Root m/z 43 | IX | 31.65 |  |  |  | IV |
| Stem m/z 123 | V | 63.33 | 9 | Stem specific | 2.33 | I |
| Stem m/z 125 |  | 64.86 |  |  |  | III |
| Stem m/z 138 |  | 65.66 |  |  |  | I |
| Root m/z 154 | V | 120.39 |  |  |  | II |
| Stem m/z 144 | VI | 41.58 |  |  |  | III |
| Root m/z 154 | VI | 61.07 | 10 | Root and stem | 26.72 | II |
| Root m/z 114 |  | 63.62 |  |  |  | IV |
| Root m/z 150 |  | 64.62 |  |  |  | II |
| Root m/z 57 |  | 64.62 |  |  |  | IV |
| Root m/z 144 |  | 65.62 |  |  |  | IV |
| Root m/z 154 |  | 65.62 |  |  |  | II |
| Root m/z 97 |  | 65.62 |  |  |  | IV |
| Root Lignin |  | 66.68 |  |  |  | II |
| Root m/z 170 |  | 66.68 |  |  |  | II |
| Root m/z 41 |  | 66.68 |  |  |  | IV |
| Root m/z 60 |  | 66.68 |  |  |  | IV |
| Root m/z 73 |  | 66.68 |  |  |  | IV |
| Stem m/z 170 |  | 68.68 |  |  |  | I |
| Stem m/z 60 |  | 68.68 |  |  |  | III |
| Stem m/z 97 |  | 68.68 |  |  |  | III |
| Stem m/z 73 |  | 68.69 |  |  |  | III |
| Root m/z 123 |  | 69.69 |  |  |  | II |
| Root m/z 85 |  | 70.41 |  |  |  | IV |
| Root SG |  | 71.16 |  |  |  | II |
| Root m/z 137 |  | 71.16 |  |  |  | II |
| Root m/z 98 |  | 71.16 |  |  |  | IV |
| Stem lignin |  | 71.16 |  |  |  | I |
| Stem m/z 85 |  | 71.16 |  |  |  | III |
| Stem m/z 125 |  | 72.80 |  |  |  | III |
| Stem m/z 57 |  | 72.80 |  |  |  | III |
| Stem m/z 98 |  | 73.02 |  |  |  | III |
| Stem m/z 123 |  | 79.80 |  |  |  | I |
| Stem SG |  | 80.74 |  |  |  | I |
| Stem lignin |  | 82.47 |  |  |  | I |
| Stem m/z 73 |  | 82.47 |  |  |  | III |
| Stem m/z 114 |  | 87.79 |  |  |  | III |
| Stem m/z 97 |  | 87.79 |  |  |  | III |
| Stem m/z 144 | VI | 102.58 | 11 | Stem specific | 5.00 | III |
| Stem m/z 123 |  | 107.58 |  |  |  | I |
| Root m/z 154 | VII | 8.62 |  |  |  | II |
| Root m/z 157 | VII | 38.25 | 12 | Root specific | 8.89 | II |
| Root m/z 158 |  | 46.15 |  |  |  | II |
| Root m/z 172 |  | 47.23 |  |  |  | II |
| Root m/z 41 |  | 47.23 |  |  |  | IV |
| Stem m/z 168 | VII | 91.31 | 13 | Stem specific | 10.60 | I |
| Stem m/z 170 |  | 91.31 |  |  |  | I |
| Stem m/z 125 |  | 94.91 |  |  |  | III |
| Stem m/z 57 |  | 95.91 |  |  |  | III |
| Stem m/z 85 |  | 101.91 |  |  |  | III |
| Stem m/z 138 | VIII | 4.74 | 14 | Root and stem | 20.00 | I |
| Root m/z 154 |  | 7.78 |  |  |  | II |
| Root m/z 98 |  | 9.78 |  |  |  | IV |
| Root m/z 114 |  | 10.10 |  |  |  | IV |
| Root m/z 123 |  | 13.04 |  |  |  | II |
| Root m/z 150 |  | 13.04 |  |  |  | II |
| Stem m/z 168 |  | 13.04 |  |  |  | I |
| Root m/z 137 |  | 14.04 |  |  |  | II |
| Root m/z 144 |  | 16.57 |  |  |  | IV |
| Root m/z 170 |  | 16.57 |  |  |  | II |
| Root m/z 172 |  | 16.57 |  |  |  | II |
| Root m/z 60 |  | 16.57 |  |  |  | IV |
| Root m/z 73 |  | 16.57 |  |  |  | IV |
| Root m/z 97 |  | 16.57 |  |  |  | IV |
| Root m/z 198 |  | 20.24 |  |  |  | II |
| Stem SG |  | 24.74 |  |  |  | I |
| Stem m/z 97 | VIII | 47.51 |  |  |  | III |
| Root m/z 58 | X | 20.90 |  |  |  | IV |
| Root SG | X | 48.51 | 15 | Root specific | 1.00 | II |
| Root m/z 157 |  | 49.51 |  |  |  | II |
| Root m/z 138 | X | 66.44 | 16 | Root and stem | 13.71 | II |
| Root m/z 94 |  | 66.66 |  |  |  | II |
| Stem m/z 94 |  | 66.66 |  |  |  | III |
| Root Lignin |  | 67.72 |  |  |  | II |
| Root m/z 114 |  | 67.72 |  |  |  | IV |
| Root m/z 144 |  | 67.72 |  |  |  | IV |
| Root m/z 85 |  | 67.72 |  |  |  | IV |
| Root m/z 97 |  | 67.72 |  |  |  | IV |
| Root m/z 98 |  | 67.72 |  |  |  | IV |
| Stem m/z 138 |  | 67.72 |  |  |  | I |
| Stem m/z 41 |  | 72.19 |  |  |  | III |
| Stem m/z 43 |  | 72.19 |  |  |  | III |
| Stem m/z 99 |  | 76.47 |  |  |  | III |
| Stem lignin |  | 80.15 |  |  |  | I |
| Stem m/z 73 | X | 94.63 | 17 | Root and stem | 13.30 | III |
| Root m/z 85 |  | 100.63 |  |  |  | IV |
| Stem m/z 94 |  | 100.63 |  |  |  | III |
| Root m/z 144 |  | 101.63 |  |  |  | IV |
| Root m/z 97 |  | 101.63 |  |  |  | IV |
| Root m/z 73 |  | 103.63 |  |  |  | IV |
| Root m/z 94 |  | 103.63 |  |  |  | II |
| Root m/z 60 |  | 104.63 |  |  |  | IV |
| Root m/z 114 |  | 106.05 |  |  |  | IV |
| Root m/z 150 |  | 106.05 |  |  |  | II |
| Root m/z 58 |  | 106.05 |  |  |  | IV |
| Stem m/z 138 |  | 107.05 |  |  |  | I |
| Stem m/z 114 |  | 107.93 |  |  |  | III |
| Stem m/z 144 |  | 107.93 |  |  |  | III |
| Stem m/z 97 | X | 115.52 |  |  |  | III |
| Root Lignin | X | 130.16 | 18 | Root specific | 0.00 | II |
| Root m/z 123 |  | 130.16 |  |  |  | II |
| Root m/z 137 |  | 130.16 |  |  |  | II |
| Root m/z 170 |  | 130.16 |  |  |  | II |
| Root m/z 98 |  | 130.16 |  |  |  | IV |
| Root m/z 58 | XI | 17.68 | 19 | Root and stem | 0.00 | IV |
| Stem m/z 119 |  | 17.68 |  |  |  | I |
| Root m/z 184 | XII | 15.40 | 20 | Root specific | 3.39 | II |
| Root m/z 58 |  | 15.40 |  |  |  | IV |
| Root m/z 125 |  | 18.79 |  |  |  | IV |
| Stem m/z 123 | XII | 37.61 | 21 | Root and stem | 3.00 | I |
| Root m/z 158 |  | 39.61 |  |  |  | II |
| Root m/z 98 | XIII | 35.94 | 22 | Root specific | 2.99 | IV |
| Root m/z 85 |  | 38.94 |  |  |  | IV |
| Root SG | XIII | 50.39 |  |  |  | II |
| Root m/z 125 | XIV | 1.00 | 23 | Root specific | 5.66 | IV |
| Root m/z 170 |  | 3.00 |  |  |  | II |
| Root m/z 184 |  | 3.00 |  |  |  | II |
| Root m/z 43 |  | 3.00 |  |  |  | IV |
| Root m/z 144 |  | 4.66 |  |  |  | IV |
| Root m/z 60 |  | 4.66 |  |  |  | IV |
| Root m/z 98 |  | 4.66 |  |  |  | IV |
| Root Lignin |  | 5.66 |  |  |  | II |
| Root m/z 154 |  | 5.66 |  |  |  | II |
| Root m/z 73 |  | 5.66 |  |  |  | IV |
| Root m/z 85 |  | 5.66 |  |  |  | IV |
| Root m/z 114 |  | 6.66 |  |  |  | IV |
| Root m/z 58 |  | 6.66 |  |  |  | IV |
| Stem m/z 150 | XIV | 11.54 |  |  |  | I |
| Stem m/z 154 | XIV | 13.22 |  |  |  | I |
| Stem m/z 125 | XIV | 14.22 |  |  |  | III |
| Stem m/z 154 | XIV | 27.81 | 24 | Stem specific | 4.91 | I |
| Stem m/z 198 |  | 32.72 |  |  |  | I |
| Stem m/z 94 |  | 32.72 |  |  |  | III |
| Stem SG | XIV | 50.37 |  |  |  | I |
| Stem m/z 154 | XV | 21.12 |  |  |  | I |
| Root m/z 41 | XV | 56.31 |  |  |  | IV |
| Root m/z 168 | XV | 71.15 | 25 | Root and stem | 7.02 | II |
| Root m/z 138 |  | 78.15 |  |  |  | II |
| Stem m/z 168 |  | 85.17 |  |  |  | I |
| Root m/z 172 | XVI | 24.79 |  |  |  | II |
| Root m/z 184 | XVI | 36.99 |  |  |  | II |
| Root m/z 114 | XVI | 49.87 | 26 | Root and stem | 0.00 | IV |
| Root m/z 94 |  | 49.87 |  |  |  | II |
| Stem m/z 97 |  | 49.87 |  |  |  | III |
| Stem m/z 99 | XVI | 49.87 |  |  |  | III |
| Root m/z 138 | XVIII | 11.00 |  |  |  | II |
| Stem m/z 123 | XVIII | 39.45 | 27 | Root and stem | 3.23 | I |
| Root m/z 94 |  | 42.69 |  |  |  | II |
